# Identification of consensus RNA secondary structures using suffix arrays

**DOI:** 10.1186/1471-2105-7-244

**Published:** 2006-05-05

**Authors:** Mohammad Anwar, Truong Nguyen, Marcel Turcotte

**Affiliations:** 1 School of Information Technology and Engineering, University of Ottawa, Ottawa, Ontario, Canada

## Abstract

**Background:**

The identification of a consensus RNA motif often consists in finding a conserved secondary structure with minimum free energy in an ensemble of aligned sequences. However, an alignment is often difficult to obtain without prior structural information. Thus the need for tools to automate this process.

**Results:**

We present an algorithm called Seed to identify all the conserved RNA secondary structure motifs in a set of unaligned sequences. The search space is defined as the set of all the secondary structure motifs inducible from a seed sequence. A general-to-specific search allows finding all the motifs that are conserved. Suffix arrays are used to enumerate efficiently all the biological palindromes as well as for the matching of RNA secondary structure expressions.

We assessed the ability of this approach to uncover known structures using four datasets. The enumeration of the motifs relies only on the secondary structure definition and conservation only, therefore allowing for the independent evaluation of scoring schemes. Twelve simple objective functions based on free energy were evaluated for their potential to discriminate native folds from the rest.

**Conclusion:**

Our evaluation shows that 1) support and exclusion constraints are sufficient to make an exhaustive search of the secondary structure space feasible. 2) The search space induced from a seed sequence contains known motifs. 3) Simple objective functions, consisting of a combination of the free energy of matching sequences, can generally identify motifs with high positive predictive value and sensitivity to known motifs.

## Background

The history of molecular biology is punctuated by a series of discoveries demonstrating the surprising breadth of biological roles of RNAs. The repertoire of known non-protein coding RNAs (ncRNAs) has grown rapidly [1]. The housekeeping roles of RNAs, such as those of tRNA, rRNA, RNaseP, snRNA and snoRNA, were discovered early. While in the recent years, it became clear that RNAs also have important regulatory functions. Examples include microRNAs, which regulate the expression of protein genes by targeting a complementary region of their mRNAs. MicroRNAs constitute one of the most abundant classes of regulatory molecules, and are key to many developmental processes [2]. Several discoveries collectively demonstrate that untranslated messenger RNAs can sense the level of metabolites, and modulate the expression of the genes accordingly. Those RNAs are referred to as RNA sensors and riboswitches, see [3,4] for a review. Post-transcriptional regulation of gene expression often involves secondary structure elements located in the untranslated regions of mRNAs [5]. Through all those discoveries, a new understanding of gene expression regulation is emerging.

Much work has been done on predicting RNA structure rather than predicting RNA consensus motifs. The most popular approach to structure prediction is perhaps the minimum free energy approach pioneered by Zuker [6-8]. Simply, the free energy of an RNA molecule is modeled as a sum of independent contributions of cycles (so called nearest neighbour model [9]). Melting experiments are performed to determine the free energy parameters for small structures. Since the free energy can be decomposed into a sum of independent contributions, it can be solved exactly and efficiently when formulated as a dynamic programming problem [6]. Steady progress has been made, mainly through the determination of more complete and accurate sets of free energy parameters [8], and the performance of this approach is well known [10]. However, there are several reasons why free energy minimisation methods can fail.

• The lowest free energy conformation may not coincide with the native conformation. This can be due to experimental errors in determining the free energy parameters, errors due to the extrapolation of the parameters, or simply because there are numerous lowest free energy conformations, and it can be difficult to distinguish the native conformation from the others;

• Certain classes of RNA have more than one active structure. This is the case for several RNA regulatory elements termed riboswitches [3,4,11];

• The nearest neighbour model does not take into account the contributions of the cellular environment: proteins, other RNAs, metabolites and solvent. Such contributions may be particularly important for modeling regulatory elements present in the untranslated regions of mRNAs;

• Similarly, RNAs are often modified after their transcription, the modifications can play an important role while folding;

• Higher-order structures, including pseudo-knotted structures, are often not considered. For some RNAs, the lowest free energy conformation obtained when neglecting pseudo-knots will be different from that of the native conformation. However, taking into account pseudo-knots severely increases the time and space complexity of the algorithms. Finally, there is also a lack of experimental data that can be used to deduce the free energy parameters.

The accuracy of RNA secondary structure prediction can often be increased if a multiple sequence alignment is used as input; these sequences are assumed to share a common secondary structure. For example, Hofacker *et al*. have incorporated an additional term into the total energy function for taking into account covariations [12]. This approach has been implemented in the program RNAalifold. The authors have shown that the number of required input sequences is less than that of traditional covariations analyses, yet the results are superior to the implementations based on a single input sequence. Often, an alignment is not readily available. It could be that the similarity of the available sequences is too low to construct a multiple sequence alignment; consequently, knowledge about the secondary structure would be required to construct a reliable alignment. Alternatively, the common motif perhaps only represents a small portion of each sequence; and it can be discontinuous.

David Sankoff has developed recurrence equations to simultaneously fold and align RNA sequences [13] (to be more precise, the work also proposes the reconstruction of the ancestral sequence on a phylogenetic tree, a "tour de force"). In principle, the method could be used to align RNA sequences with low similarity. In practice, its time and space complexity limits its application. Dynalign is an implementation of this algorithm for two sequences [14]. It differs from the original proposal in that there are no substitution costs present in the recurrence equation. Masoumi and Turcotte recently extended this work for three input sequences [15,16]. This work has shown that 1) the average positive predictive value (PPV) is improved when using three input sequences rather than two, 2) there are fewer low PPV predictions and 3) the sensitivity is only slightly affected. Together, these two research projects suggest that using several input sequences can significantly improve the PPV of secondary structure prediction methods. However, the prohibitive time and space complexity of these algorithms limits their application to sequences that are a few hundreds nucleotides long, and approximately the same length. Indeed, the time complexity of the algorithm for three input sequences is O
 MathType@MTEF@5@5@+=feaafiart1ev1aaatCvAUfKttLearuWrP9MDH5MBPbIqV92AaeXatLxBI9gBamrtHrhAL1wy0L2yHvtyaeHbnfgDOvwBHrxAJfwnaebbnrfifHhDYfgasaacH8akY=wiFfYdH8Gipec8Eeeu0xXdbba9frFj0=OqFfea0dXdd9vqai=hGuQ8kuc9pgc9s8qqaq=dirpe0xb9q8qiLsFr0=vr0=vr0dc8meaabaqaciaacaGaaeqabaWaaeGaeaaakeaat0uy0HwzTfgDPnwy2aqeh0uy0HwzTfgDPnwy2aacfaGae8NdW=eaaa@4191@(|*S*_1_|^3^*M*^6^), where |*S*_1_| is the length of the shortest input sequence and *M *is the maximum distance between aligned nucleotides.

As a result, the identification of RNA motifs requires extensive human examination. This paper presents a new software system that allows searching exhaustively the space of RNA sequence and structure motifs, therefore assisting the identification and characterisation of consensus structures. Since the algorithm is exhaustive and independent of any scoring scheme, it is ideally suited to study the ability of objective functions to identify native folds.

## Results and discussion

### Search space

For a given input sequence, the number of valid RNA secondary structures is extremely large; exponential with respect to its length [17]. In order to make the search space more tractable, we adopt a data-driven approach. A seed sequence serves to induce a search space that is exhaustively explored for finding motifs that also match a significant fraction of all the input sequences. The search space is traversed from the most general to the most specific motif. Whenever a motif is found that is not supported (does not match the required minimum number of input sequences) the motif, and its descendants, are pruned from the search space. Herein, a motif is defined as a collection of one or more stems, where each base pair is either generic (Watson-Crick or Wobble pair) or specified (e.g. G:C). No other criteria than support and the validity of the structures are used to prune the search space.

### Algorithm

There can be several sources of noise. Firstly, the assumption that the input sequences are sharing a common structure has to be true for the majority of the input sequences. Herein, all the input sequences selected contain exactly one copy of the consensus structure. However, in general the possibility that certain sequences have been erroneously included in the input cannot be excluded. Secondly, the input perhaps consists of more than one fold family. Thirdly, some of the sequences could adopt a less conserved structure, which to be found would require relaxing the parameters of the algorithm up to a point where it becomes impractical. Hence, a user-defined level of noise is tolerated. The support is defined as the fraction of the input sequences containing a given motif; in the experiments presented here, we set the support to 70%. The main steps of the algorithm are as follows.

1. Select a seed sequence;

2. Construct the most specific motif;

3. General-to-specific search of the motif space;

4. Report the motifs.

By default, the first sequence (index 0) is used as the seed sequence. The software system provides an option to select a specific sequence as the seed sequence. Valid values are integers in the range 0 ... *k *- 1, where *k *is the number of input sequences. Selecting the shortest sequence should reduce the size of the search space, and consequently the execution time.

#### Most specific motif

The search space is induced from a seed sequence that has been selected in the first step of the algorithm. The method is described using suffix trees, however, the implementation uses suffix arrays, see Methods for further implementation details. For the exposition of the basic algorithm below, let *S *be the seed sequence and *S*^*R *^be the reverse complement of *S*.

1. Construct a generalised suffix tree for *S *and *S*^*R*^*;*

2. For every starting position *i = *1 ... *n *in *S*;

2.1. For every starting position *j *in *S*, such that *i + C<j≤i + L;*

2.1.1. Find the lowest common ancestor of *i *and *j'*, where *j' = |S| - j *+ 1 is the corresponding index in *S*^*R*^;

2.1.2. If the length of the complementary region is larger than some user-defined value then save this stem.

where *C *and *L *are user-defined constraints specifying the minimum and maximum distance between the 5' end and the 3' end of a stem. The basic algorithm is extended in two ways. First, up to *e *mismatches per stem are allowed. This involves adding an inner loop, executed *e *times. This increases the time complexity by a factor *e*. The second extension allows for up to *m *wobble base pairs (G:U); which are handled in a similar way as mismatches. The location of each stem is recorded to be used in the later stages of the algorithms. By using suffix arrays and range minimum query, the stems are enumerated in O
 MathType@MTEF@5@5@+=feaafiart1ev1aaatCvAUfKttLearuWrP9MDH5MBPbIqV92AaeXatLxBI9gBamrtHrhAL1wy0L2yHvtyaeHbnfgDOvwBHrxAJfwnaebbnrfifHhDYfgasaacH8akY=wiFfYdH8Gipec8Eeeu0xXdbba9frFj0=OqFfea0dXdd9vqai=hGuQ8kuc9pgc9s8qqaq=dirpe0xb9q8qiLsFr0=vr0=vr0dc8meaabaqaciaacaGaaeqabaWaaeGaeaaakeaat0uy0HwzTfgDPnwy2aqeh0uy0HwzTfgDPnwy2aacfaGae8NdW=eaaa@4191@(*n *+ *emLn*) time. Similar ideas have been proposed by Gusfield [18], for suffix trees only.

#### General-to-specific search

The search algorithm consists of three distinct phases: initialisation, instantiation and composition. During the first phase, the algorithm initialises a queue of open nodes to contain structural motifs (see below). The motifs have been derived from the selected seed sequence. Only the motifs that have a minimum support, i.e. that also match other sequences from the input set, are part of the queue. Structural motifs have no base pair instantiated.

**Figure 1:**
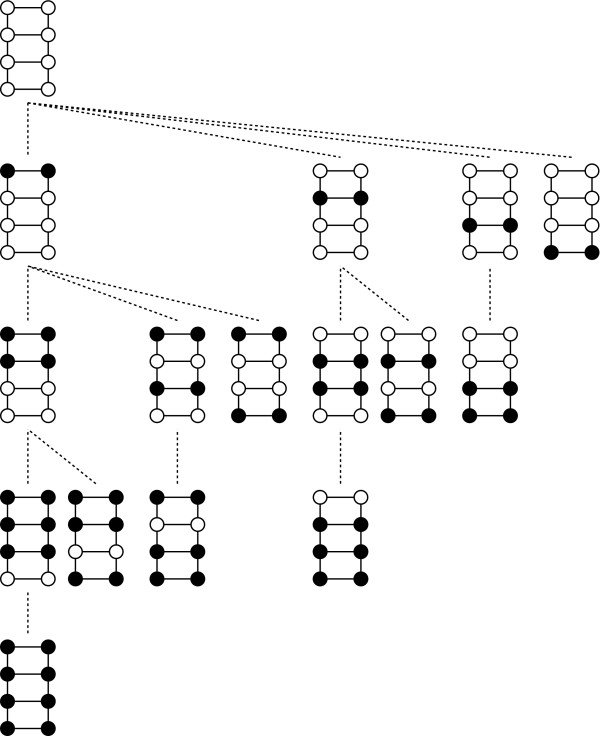
**Sequence instantiation process**. Schematic illustration of the sequence instantiation process. Open circles correspond to generic base pairs, N:N', while the filled circles represent specific base pair, A:U, U:A, C:G, G:C, G:U and U:G.

In the second phase, all the possible sequence instantiations for every motif of the open queue are considered. Systematically and exhaustively, all the base pairs of every stem motif in the open queue are replaced by the actual base pair that occurs in the seed sequence. This information is readily available since the location of every stem within the seed sequence has been saved. Each newly created instance is matched against the remaining sequences. Only the motifs that have a minimum level of support are added at the rear of the queue. Figure 1 illustrates this process for a single stem. Progressively, all the possible instantiations are validated. This is done systematically so that the same instantiation is never considered twice. This behaviour is controlled using the parameter max_fixed_pos. Setting max_fixed_pos to 0 eliminates sequence motifs from the search space. At the end of the second phase, the open queue contains a mixture of structural, partially and fully instantiated motifs, all consisting of a single stem segment. Finally, the third phase consists of creating multi-stems motifs by selecting and composing two motifs at a time from the open queue. The composition of two motifs is dictated by their occurrence within the seed sequence. Given two motifs, there are two possible relationships. One motif follows the other or one motif is nested within the other. The seed sequence is used to determine which relationship to use and to calculate the distances. This process creates helices with bulges and interior loops as well as multi-branch structures. Motifs that are structurally invalid (because they overlap in the sequence space) or that do not have the required minimum support are discarded. The open queue now contains a mixture of single and multi-stems motifs, that are structural, partially or fully instantiated.

#### Example of the execution of Seed

**Figure 2:**
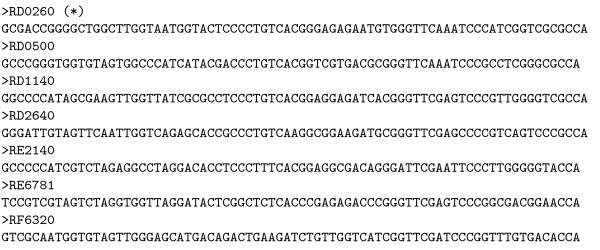
**First step of the Seed algorithm**. Schematic illustration of the execution of the first step of the algorithm. In this example there are seven input sequences and RD0260 has been selected to be the seed sequence.

**Figure 3:**
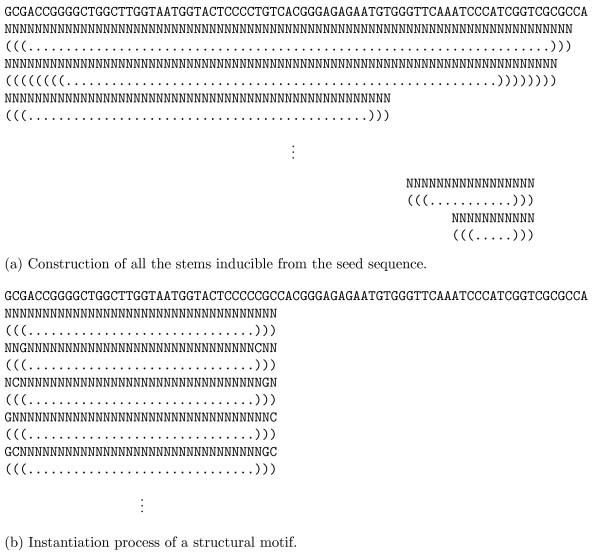
**Second step of the Seed algorithm**. Schematic illustration of the execution of the second step of the algorithm. (a) Construction of all the stems inducible from the seed sequence. (b) Instantiation process of a structural motif.

**Figure 4:**
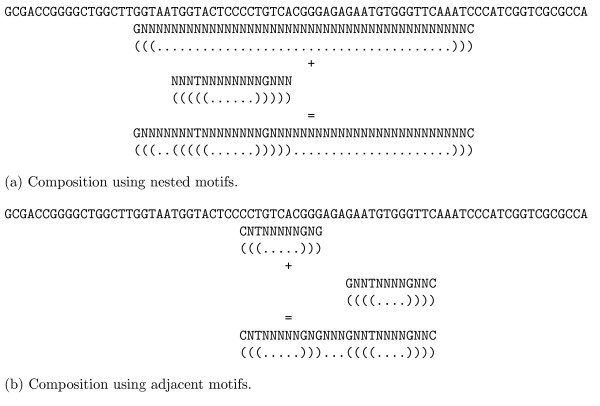
**Third step of the Seed algorithm**. Schematic illustration of the execution of the third step of the algorithm. (a) Composition using nested motifs. (b) Composition using adjacent motifs.

The algorithm consists of three main steps: the selection of the seed sequence, the construction of the most specific motif, and the general-to-specific search of the motif space. To illustrate the execution of Seed, we use the tRNA dataset. In the first step, the software system selects a seed sequence. The default is to select the first sequence (index 0), see Figure 2. The selected sequence is used for constructing a set of building blocks that will be combined in the third step of the algorithm to produce complex motifs. First, all the complementary regions are enumerated, see Figure 3(a). Next, each complementary region is used to create specific motifs containing one or more base pairs with known identity, see Figure 3(b). Each time a new motif is created, Seed matches it against the remaining (*k *- 1) sequences. A motif is saved, and will be used to create more complex motifs, only if its support is sufficient. Finally, the third step of the algorithm is the general-to-specific search of the motif space. Elements of the open queue are combined with motifs created in the second step. Elements are combined using information from the seed sequence. These elements can be nested or adjacent in the seed sequence, see Figures 4(a) and 4(b). Again, a motif is saved, and will be used to create more complex motifs, only if its support is sufficient.

#### Secondary structure expression matcher

For all three phases of the search algorithm, the newly created motifs are matched against all the input sequences, in order to determine their level of support.

Since the motifs are repeatedly matched against a fixed set of sequences, it is advantageous to pre-process the input sequences to speed-up the matching operations. We introduce an algorithm for matching secondary structure expressions. The basic idea is to "thread" a secondary structure expression onto the suffix tree (suffix array) of the input sequences. This means simultaneously traversing the expression, from its 5' end, and the suffix tree, starting from its root.

The main steps of this algorithm are as follows. First, build a suffix tree for each input sequence (or build a generalised suffix tree). Then, match the characters of the secondary structure expression along the unique path in the suffix tree until either 1) the end of the secondary structure expression is reached, 2) the end of a branch is reached, 3) a mismatch is found, or 4) the secondary structure expression contains a joker (don't care symbol, any base type should be allowed).

In the former case, every leaf of the subtree below the last match represents the starting location of an occurrence. For cases 2 and 3, this is a failure and the algorithm must restart from the last branch point (see below), if there are no more branch points, this means the expression does not occur in the input sequence.

Case 4, there are three situations to consider: the joker occurs in a loop region, the joker occurs in the 5' end region of a stem, or it occurs in the 3' end region of a stem.

First, let us assume that the matching character was found along an edge of the tree but it was not the last letter of the label of that edge (the case of the last letter of an edge will be dealt with separately). The first situation is easy to handle; the next character along this path is accepted. Second situation, a joker has been found in a 5' end region of a stem. The algorithm accepts the next symbol along the current path, and pushes that symbol onto a stack. Next and third situation, a joker is encountered in a 3' end region of a stem, the top of the stack contains the base that occurred at the 5' position of the pair, if the next character along the current path inside the tree is its complement then the top element of the stack is discarded and the algorithm continues, otherwise this is a failure and the algorithm restarts from an earlier branch point, or stops, indicating a failure. Whenever the algorithm backtracks to this point, it pushes back the discarded element onto the stack. When the end of a secondary structure expression is reached (case 1) the stack must also be empty, otherwise, the expression is not valid.

Finally, whenever a joker is found and the previous match occurred at the end of a label, the algorithm has now reached an internal node of the suffix tree. All the outgoing edges of this internal node represent all the different ways to continue matching the expression. The algorithm is therefore applied recursively for all the outgoing edges. The system stack serves to memorise all these branch points.

The algorithm can answer two specific questions: 1) does this secondary structure expression occur in this input string? 2) how many occurrences of this expression are there? For the decision question, the algorithm stops whenever the end of the secondary structure expression is found. For the latter question, all the remaining branch points must be explored, and all the leaves of the subtree below the node where the last character of the expression was matched must be visited in order to count the number of occurrences, or simply to report them.

### Evaluation

Seed is a framework for finding conserved RNA motifs in a set of unaligned sequences. As such, it allows for the independent study of functions for ranking the motifs *a posteriori*.

Information content has often been used in the context of sequence pattern discovery. Accordingly, we include a function, *TInfo*, consisting of the sum of the information content contributions from unpaired and paired regions. Shannon uncertainty (*H*) is calculated for each loop position and is subtracted from the maximum uncertainty possible, to give the information content (in bits). *H *= -∑*P*_*i*_log_2 _*P*_*i *_summed over each base pair (*i *= A, U/T, G, C), where the observed nucleotide frequencies of each base *i *from the input sequences is used to estimate *P*_*i*_. A nucleotide in a stem is base paired to its partner, which increases the information content relative to an unpaired nucleotide in a loop. The resulting loop and stem information contents are added to calculate the total information content.

Work on simultaneous alignment and structure prediction of RNA sequences [14-16] suggests that a (linear) combination of the free energy of multiple input sequences, when folded onto a common structure, can help circumvent limitations of the nearest neighbour model, and effectively identify native structures. We consider here several simple functions combining the free energy of all or some of the matches of a given motif. These functions are: *TLeft, NTLeft, TFirst, NTFirst, TSum, NTSum, TBest, NTBest, TWorst, NTWorst, TAvg *and *NTAvg*.

Each motif matches at least **min_support **× *k *sequences, and up to *k *sequences, by construction. Certain motifs will occur more than once in a given sequence. Thus, there are several ways to calculate the free energy score for a given motif and set of matches. Furthermore, the secondary structure expression matcher, which we developed for this work, traverses the suffix array of the input sequences, rather than the input sequences themselves. Consequently, the matches are reported in lexicographic order. The execution time can be slightly reduced by taking into account the free energy of the first reported match, rather than finding the best or leftmost one.

For a given motif, *TLeft *is the sum of the free energy of its leftmost occurrence in each sequence. *TFirst *has a slightly different definition. *TFirst *is the sum of the free energy of the first occurrence reported by the matcher in each sequence. This function was considered since it can slightly reduce the execution time. *TSum *is the sum of the free energy of all the occurrences in all the matching sequences. *TBest *is the sum of the lowest free energy match in each sequence. *TAvg *is the sum of the average free energy of all the matches per sequence. *TWorst *is the sum of the highest free energy match from each sequence. Finally, normalised variants of these scores are obtained by dividing each score by the number of matched sequences or total number of matches for *TSum; *the resulting scores are noted with the letter *N *followed by the name of the base score.

We used two criteria to compare the scoring functions. We measured the correlation coefficient for each function against the Matthews Correlation Coefficient (MCC, see Methods for a definition of these performance measures). Clearly, a perfectly correlated function would allow selecting the consensus structure with the best MCC. However, since the primary objective of the scoring functions is to order the consensus structures from best to worst, rather than modeling the free energy, we also used the ranking-based evaluation measures recently proposed by Rosset *et al*. [19]. The ranking statistics describe the relationship between scoring functions and the MCC performance measure. Each consensus structure is sorted in increasing order of energy scores (decreasing for information content) and assigned unique ranks. For each pair of motif making an incorrect ordering of MCC, the weighted difference of their ranks is calculated. This is then transformed into the range [-1,1].

Table 3 presents the results for all six experiments. The weighted (ρ^
 MathType@MTEF@5@5@+=feaafiart1ev1aaatCvAUfKttLearuWrP9MDH5MBPbIqV92AaeXatLxBI9gBaebbnrfifHhDYfgasaacH8akY=wiFfYdH8Gipec8Eeeu0xXdbba9frFj0=OqFfea0dXdd9vqai=hGuQ8kuc9pgc9s8qqaq=dirpe0xb9q8qiLsFr0=vr0=vr0dc8meaabaqaciaacaGaaeqabaqabeGadaaakeaaiiGacuWFbpGCgaqcaaaa@2E83@) rankings are very similar for all the objective functions studied; ρ^
 MathType@MTEF@5@5@+=feaafiart1ev1aaatCvAUfKttLearuWrP9MDH5MBPbIqV92AaeXatLxBI9gBaebbnrfifHhDYfgasaacH8akY=wiFfYdH8Gipec8Eeeu0xXdbba9frFj0=OqFfea0dXdd9vqai=hGuQ8kuc9pgc9s8qqaq=dirpe0xb9q8qiLsFr0=vr0=vr0dc8meaabaqaciaacaGaaeqabaqabeGadaaakeaaiiGacuWFbpGCgaqcaaaa@2E83@ varies from -1 to 1, where a -1 score signifies an anti-correlation while +1 means a perfect positive correlation. It is interesting to note that the objective function having the highest ρ^
 MathType@MTEF@5@5@+=feaafiart1ev1aaatCvAUfKttLearuWrP9MDH5MBPbIqV92AaeXatLxBI9gBaebbnrfifHhDYfgasaacH8akY=wiFfYdH8Gipec8Eeeu0xXdbba9frFj0=OqFfea0dXdd9vqai=hGuQ8kuc9pgc9s8qqaq=dirpe0xb9q8qiLsFr0=vr0=vr0dc8meaabaqaciaacaGaaeqabaqabeGadaaakeaaiiGacuWFbpGCgaqcaaaa@2E83@ value is *NTFirst*. This function is the fastest to compute since the matcher stops after locating the first occurrence of the motif in each input sequence. The first occurrence is also the one that comes first in lexicographic order. Thus, these occurrences are perhaps more similar in terms of sequence. This is consistent with the observation that a linear combination of the free energy of matches and the information content outperforms either of these two scores alone (data not shown). The other objective functions are biased toward finding the leftmost, largest number of occurrences, best or worst free energy matches. The bias of *NTFirst *is perhaps more difficult to rationalise. However, since *NTFirst *is fast to compute and has the highest ρ^
 MathType@MTEF@5@5@+=feaafiart1ev1aaatCvAUfKttLearuWrP9MDH5MBPbIqV92AaeXatLxBI9gBaebbnrfifHhDYfgasaacH8akY=wiFfYdH8Gipec8Eeeu0xXdbba9frFj0=OqFfea0dXdd9vqai=hGuQ8kuc9pgc9s8qqaq=dirpe0xb9q8qiLsFr0=vr0=vr0dc8meaabaqaciaacaGaaeqabaqabeGadaaakeaaiiGacuWFbpGCgaqcaaaa@2E83@ value, it has been used for the analysis of the results for all six experiments.

### Experiments

We present the results of six experiments: HSL3 01, IRE 01, HSL3 02, IRE 02, tRNA and 5S. See Table 1 for the statistics, and Methods section for the description of the datasets used. The first four tests were designed to evaluate the suitability of this approach to identify automatically conserved stem-loop structures. While the last two sets were created to study the performance of the method on more complex secondary structures.

The first dataset consists of 28 3' UTR histone mRNAs that are known to contain a six base stem and four base loop structure. This stem-loop structure plays several roles, including enhancing the translation of histone mRNAs [20].

In the first experiment, HSL3 01, strict parameters were used to verify the presence of native folds in the search space. More precisely, we searched for a consensus structure containing exactly one stem segment made of at least six base pairs. The maximum distance for any two nucleotides involved in a base pair was set to 30. With these options, Seed finds two distinct structural motifs. Since all the base pair instantiations are also considered, the total number of motifs is 65, see Table 1. All but four have a Matthews correlation coefficient score of 100%, i.e. both PPV and sensitivity are 100%.

**Figure 5:**
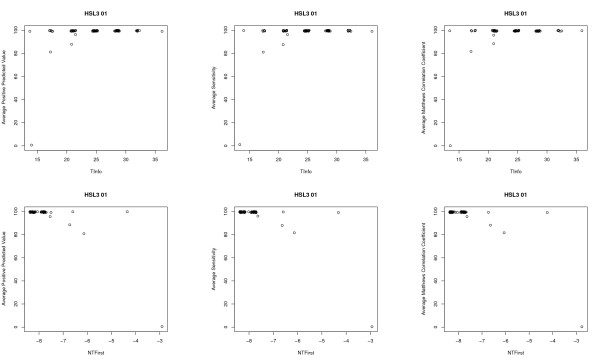
**Performance diagrams for the HSL3 01 experiment**. *TInfo *(top), *NTFirst *(bottom) scores against the PPV, sensitivity and Matthews Correlation Coefficient for the HSL3 dataset. Each data point represents the predicted rank (according to *TInfo *and *NTFirst *respectively) and average performance index (PPV, sensitivity or MCC) for a consensus structure. For better visualisation, random noise was introduced so that the data points do not overlap.

The relationship between the information content (*TInfo*) and the performance index for this experiment is characteristic of the other experiments as well. Motifs with a high information content also have good PPV and sensitivity, see Figure 5. However, there are low information content motifs that have high PPV and sensitivity. The highest correlation is observed for *NTLeft*, see Table 2. For all twelve functions, the "best" scoring motif also has the best PPV and sensitivity. The average number of matches per motif per sequence varies from 1 to 5, with an average of 1.2 matches per sequence.

**Figure 6:**
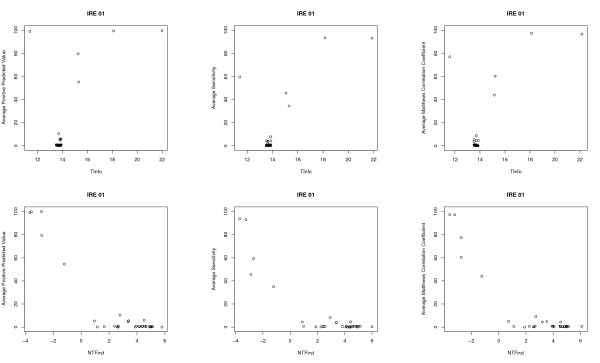
**Performance diagrams for the IRE 01 experiment**. *TInfo *(top) *NTFirst *(bottom) scores against the PPV, sensitivity and Matthews Correlation Coefficient for the IRE dataset.

The second dataset consists of 14 5' UTR sequences that are known to contain an IRE motif. In this experiment (IRE 01), we searched for a structure consisting of one or two segments of at least three base pairs each; notice that one or two stem-loop structures could be formed with these options. The maximum distance for the nucleotides of a base pair was set to 30. A total of 32 motifs were found, see Table 1, three of which have 100% PPV and more than 90% sensitivity. The number of matches per sequence varies from 1.1 to 3.9, with an average number of 1.9 matches per sequence. *TFirst *is the scoring function with the best correlation to MCC. Figure 6 illustrates the separation of native folds from the rest achieved using *NTFirst *for this experiment.

**Figure 7:**
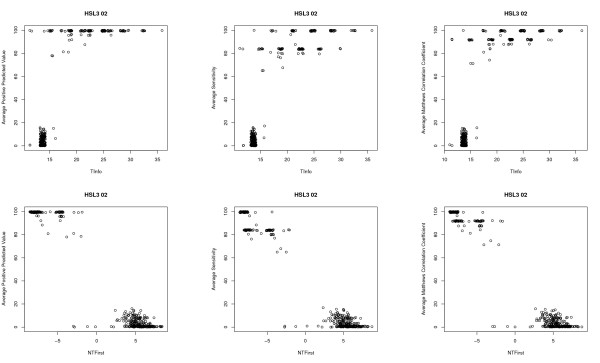
**Performance diagrams for the HSL3 02 experiment**. *TInfo *(top) and *NTFirst *(bottom) scores against the PPV, sensitivity and Matthews Correlation Coefficient for the HSL3 dataset.

Next, we reran the first two experiments with less restrictive parameters, suitable for finding stem-loop structures in general. In particular, no restrictions were imposed for the maximum number of segments that could be used to form a motif, except that the maximum distance for the elements of a pair should be 30 nucleotides. A total of 357 motifs were found for the HSL3 dataset, see HSL3 02 in Table 1. Nearly one third of the conserved motifs have a PPV of 100%. *NTWorst *has the best correlation to MCC; all the correlation scores are -0.95 or better. For this experiment, the free energy scores are particularly effective for separating the high PPV/sensitivity motifs from the rest, see Figure 7.

**Figure 8:**
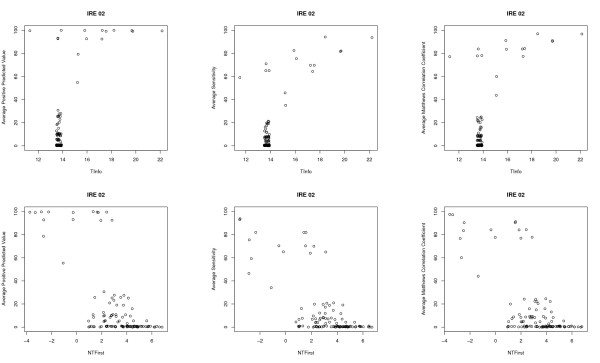
**Performance diagrams for the IRE 02 experiment**. *TInfo *(top) and *NTFirst *(bottom) scores against the PPV, sensitivity and Matthews Correlation Coefficient for the IRE dataset.

Similarly, the IRE experiment (IRE 02) was carried out relaxing the parameters. No restrictions were imposed on the maximum number of segments, the minimum size of the segments was three base pairs, and the maximum distance between the elements of a base pair was set to 30. A total of 110 motifs were found, see Table 1. Of the scoring schemes based on free energy, *NTWorst *has the best correlation to MCC. However, the magnitude of the correlation for the *TInfo *score is higher than that of *NTWorst*. The structural motifs with the highest MCC score rank first using *NTFirst*, see Figure 8.

**Figure 9:**
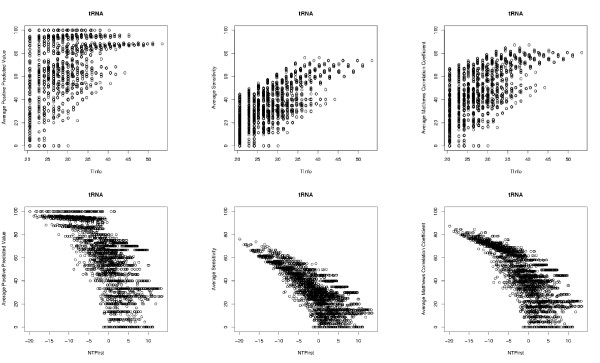
**Performance diagrams for the tRNA experiment**. *TInfo *(top) and *NTFirst *(bottom) scores against the PPV, sensitivity and Matthews Correlation Coefficient for the tRNA dataset.

**Figure 11:**
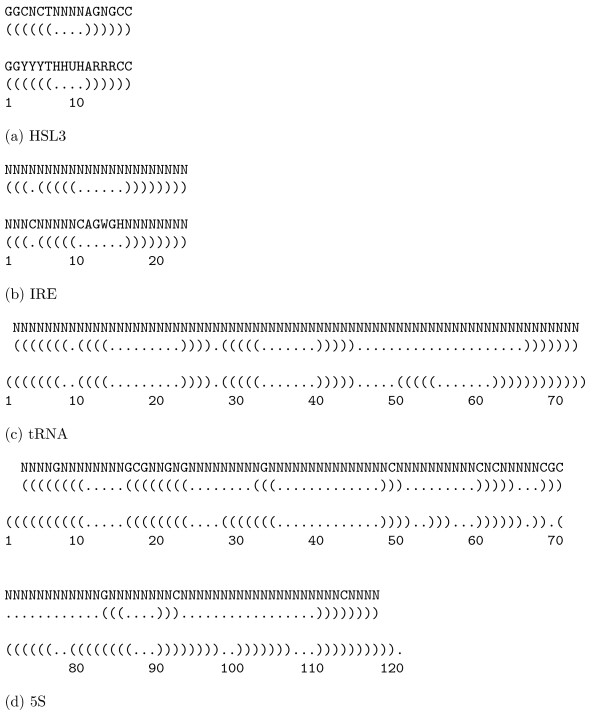
**Lowest free energy motifs**. Dot-bracket notation of the predicted (top) and reference (bottom) structures. A dot '.' represents an unpaired nucleotide. A base pair is represented as a pair of opening and closing brackets, '(' and ')'. Single letter codes: H = not G, Y = C or T/U, R = A or G, W = A or U.

We also studied structures that are more complex. The next dataset consists of 7 tRNA sequences representing diverse levels of difficulties for MFOLD, see Table 4. In order to discover multiple stem structures, we placed no restrictions on the maximum distance between elements of a base pair. Also, the length of the unpaired regions in matched sequences was allowed to vary by one nucleotide for added flexibility. The size of the output was reduced by reporting only the motifs consisting of three or more segments (complementary regions). A total of 5,518 motifs were found. *NTLeft *gave the highest correlation to MCC (-0.834). The tRNA structures have been used as a benchmark for the development of minimum free energy methods. Perhaps the free energy models are particularly proficient for this class of RNAs. Indeed, it is interesting to observe the high degree of correlation of *NTFirst *to MCC, particularly for the left tail of the distribution, see Figure 9, 10. The motif with the highest *NTFirst *score has 16 base pairs, 100% PPV, 76.2% sensitivity. It matches 5 of the 7 input sequences. Figure 11 shows the lowest free energy motifs.

The last experiment was carried out on 7 5S ribosomal RNA sequences. The sequences represent diverse levels of difficulty for MFOLD, see Table 5. The same options as above (tRNA experiment) were used. A total of 364,505 consensus motifs were found representing 24,645 distinct structures. The maximum sensitivity achieved is much lower than for the previous experiments. *TSum *has the highest correlation to MCC. Better correlation scores are observed when focusing on motifs consisting of 15 or more base pairs. The correlation scores between *TSum *and *AvgPPV, AvgSensitivity *and *AvgMCC *are -0.783, -0.838 and -0.823, respectively. The PPV of the "best" motif according to *NTFirst *is high, on average 85%. However, there are many motifs with high PPV, 100%, for the intermediate *NTFirst *values.

For comparison, we ran RNAProfile (version 2.2) on the same four datasets. This is a recently developed algorithm for finding conserved secondary structure motifs in unaligned RNA sequences [21]. Several experiments were ran, varying in small increments the region parameters (minimum l, and maximum L). The number of hairpins (H) sought was also specified. The combinations of (parameters) values producing the best performance were used for comparison (HSL3: H = 1, l = 16, L = 30; IRE: H = 1, l = 20, L = 40; tRNA: H = 3, l = 60, L = 78; 5S: H = 2, l = 90, L = 120). The number of profiles kept at each step was 100. The number of profiles reported were HSL3 = 100, IRE = 86, tRNA = 45 and 5S = 71.

On the HSL3 data set, both algorithms identified motifs with 100% PPV and sensitivity. In case of IRE, the performance of both systems were comparable. Specifically, the PPV/sensitivity of the structures predicted by RNAProfile ranged from 71.4–100/62.5–100%, excluding one prediction which had no true positives. The motif predicted by Seed matched 10 out of 13 sequences with PPV/sensitivity ranging from 100–100/80–100%. In case of complex structures, tRNA and 5S, Seed outperformed RNAProfile in terms of PPV. For the tRNA data set, the PPV/sensitivity range for RNAProfile and Seed were found to be 25–100/23.8–90.5% and 100–100/76.2–76.2%, respectively. RNAProfile failed to predict the overall Y shape of the 5S RNAs. The minimum and maximum PPV/sensitivity of motifs predicted by RNAProfile was found to be 0–79.3/0–60.5% whereas by Seed it is 77.3–86.4/44.7–50%.

For all six experiments, the diagrams, correlation coefficients and ranking statistics support the use of free energy for ranking consensus motifs. Top-ranked motifs generally correspond to high PPV/sensitivity motifs while bottom-ranked motifs correspond to low PPV/sensitivity motifs. The diagrams suggest that motifs with positive free energy can be eliminated from the search space.

## Conclusion

We developed a combinatorial algorithm for the detection of consensus RNA secondary structure motifs in a set of unaligned sequences. Our algorithm compares favourably to existing tools, such as RNAProfile. Its ability to scale and predict more complex structures looks promising. To our knowledge, this is the first algorithm that directly attempts to exhaustively explore the space of sequence and structure motifs using suffix arrays.

One of our research objectives has been to determine if support and exclusion constraints are sufficient to make an exhaustive search feasible. The six experiments presented here indicate that 1) such search under constraints is feasible and that 2) the search space contains structures with high PPV/sensitivity. Indeed, most experiments completed in minutes using megabytes of memory on a small computer server. The search space contains motifs with high PPV, often 100%. For small motifs, such as HSL3 and IRE, the sensitivity is also high, often 100%.

We also evaluated several simple functions for ranking the motifs. For single stem structures, HSL3 and IRE datasets, motifs with high information content were also found to have high PPV and sensitivity. However, the performance of *TInfo *decreases as the complexity of the motifs sought increases. Overall, the free energy based ranking functions performed better, the average weighted ranking (ρ^
 MathType@MTEF@5@5@+=feaafiart1ev1aaatCvAUfKttLearuWrP9MDH5MBPbIqV92AaeXatLxBI9gBaebbnrfifHhDYfgasaacH8akY=wiFfYdH8Gipec8Eeeu0xXdbba9frFj0=OqFfea0dXdd9vqai=hGuQ8kuc9pgc9s8qqaq=dirpe0xb9q8qiLsFr0=vr0=vr0dc8meaabaqaciaacaGaaeqabaqabeGadaaakeaaiiGacuWFbpGCgaqcaaaa@2E83@) of *NTFirst *is 0.87, compared to 0.69 for *TInfo*.

With the free energy based functions, lowest free energy scoring motifs have a high PPV/sensitivity while highest scoring motifs have a low PPV/sensitivity. The functions display good correlation to PPV and sensitivity; the correlation to sensitivity is generally higher than to PPV. The ability of all the functions to rank the motifs is good, with ρ^
 MathType@MTEF@5@5@+=feaafiart1ev1aaatCvAUfKttLearuWrP9MDH5MBPbIqV92AaeXatLxBI9gBaebbnrfifHhDYfgasaacH8akY=wiFfYdH8Gipec8Eeeu0xXdbba9frFj0=OqFfea0dXdd9vqai=hGuQ8kuc9pgc9s8qqaq=dirpe0xb9q8qiLsFr0=vr0=vr0dc8meaabaqaciaacaGaaeqabaqabeGadaaakeaaiiGacuWFbpGCgaqcaaaa@2E83@ ranging from 0.84 to 0.87. For 5 out of 6 experiments, the top ranked motif has the highest Matthews correlation coefficient.

The use of homologous sequences has been shown to help circumvent limitations of the nearest neighbour model and improve the performance of RNA secondary structure prediction methods [14]. However, the time and space complexity of these approaches limits their application to at most three sequences [15,16]. Furthermore, the distance constraint *M *that makes these algorithms practical prevents them from finding conserved local structures. Consensus motifs offer an alternative approach taking advantage of available homologous sequences. Unlike *ab initio *methods, consensus approaches can potentially scale beyond three sequences and have the ability to uncover locally conserved motifs.

Consensus motifs can be used to define structural constraints that can be given as input to an *ab initio *method. To investigate this claim, the base pairs of the best *NTFirst *motif were used as input constraints for MFOLD, which was ran on all the sequences matching that motif. For tRNA, we observed an increase in PPV/sensitivity from 69.8/70.5 to 98.3/100.0%, see Table 4. For 5S, the PPV/sensitivity rose from 54.0/58.0 to 70.8/65.7%, see Table 5. For tRNA and 5S rRNA datasets, the structural constraints successfully eliminated some of the bad minima that prevented MFOLD from finding high PPV/sensitivity structures. The combined approach considerably increases the sensitivity of the prediction compared to predictions by Seed alone. For such applications, ranking functions should favour high PPV rather that sensitivity. This is illustrated with this last experiment where a high PPV motif was used for setting structural constraints. For the 5S dataset, the PPV/sensitivity rose to 81.0/84.2%.

Seed is designed to tolerate outlier sequences. However, if an outlier sequence is selected as the seed, this approach will fail to detect a consensus structure. Currently, the user should run the algorithm with different seed sequences and check the consistency of the results. Our research is moving in the direction of adding an outer loop to the algorithm automating this process.

Other future works on this project include developing new objective functions, taking into account insertions/deletions and the number of predicted base pairs, for example, to improve the discrimination of native folds. In all our tests, a linear combination of a free energy score and information content outperforms either of these two scores alone. This is consistent with the fact that *NTFirst *was found to be the best scoring function. Indeed, since *NTFirst *selects the motifs that come first in lexicographic order, those motifs are also expected to have similar sequences. Once the objective functions have been improved, they will be used to implement pruning rules to reduce the execution time.

Seed has the ability to predict consensus secondary structures and sequence motifs. Scoring functions based on information content will rank such motifs higher than those containing structural information only. Also, generic motifs are more likely to produce multiple matches for each sequence. The motifs with base pair identities will eliminate some of the matches. In this study, we have not evaluated sequence motifs. This is because 1) the information is not readily available for all the datasets and 2) Seed currently discovers sequence motifs for stem regions only. Future direction of this work also include automated discovery of sequence patterns in the loop regions.

The determination of consensus RNA secondary structure motifs is important for understanding the structure-function relationship and post-transcriptional regulation, as well as identifying RNA targets. Seed is a new exploratory tool that can be added to the set of tools for the analysis of consensus RNA structures.

## Availability and requirements

Seed is written in ISO C and uses extensions of the standard ISO C99. The calculation of the free energy is performed with the help of RNAlib, which is part of the Vienna RNA Package, .

The software system is distributed freely under the terms of the GNU General Public License. It can be downloaded from our web site: .

## Methods

### Preliminaries

Suffix trees and suffix arrays can be used for the efficient enumeration of the stems as well as for the matching of secondary structure expressions.

Suffix trees are a prominent data structure in computational biology, powering efficient sequence comparison and repeat finding algorithms that can be applied to genomic scale data [22,23]. A related data structure, suffix arrays [24], offers some advantages over suffix trees, namely reducing the memory requirements and easier to implement algorithms [25]. Important and recent achievements now allow use of suffix arrays everywhere suffix trees were used [25]. Those achievements are: a direct approach for the linear-time construction of the suffix array [26-28], an algorithm for finding the longest-common-prefix in linear-time [29], and simulating the bottom-up [30] and top-down [31] traversal of suffix trees, also in linear time. We used suffix arrays for the implementation of Seed but we will use suffix trees herein for clarity. Reference [25] shows the relationships between the two data structures.

A suffix tree for a text *T = t*_1 _... *t*_*n *_is a rooted labeled tree with the following characteristics.

• The edges of the tree are labeled with substrings of the text;

• Each internal node has at least two children, with the possible exception of the root of the tree;

• Any two outgoing edges of the same internal node start with a different letter;

• Every suffix of the text is spelled out on a path from the root to a leaf, and that leaf is labeled with the start position of that suffix.

Several algorithms and implementation techniques have been proposed for constructing the data structure in linear-time and space. Applications include pattern matching and repeat finding. A pattern *P *occurs in a text *T *if and only if the suffix tree of *T *contains a path (from the root of the tree) that spells *P*; this follows from the fact that *P *occurs in *T *if and only if *P *is the prefix of at least one suffix of *T*. A suffix tree exposes all the internal repeats of a text. By definition, every internal node, with the possible exception of the root, has at least two descendants. All the descendants of an internal node represent suffixes that share a common prefix, spelled out on the path from the root to that internal node. All the outgoing edges start with a different letter and represent all the different extensions of this common prefix. A generalized suffix tree is a suffix tree that contains all the suffixes of two or more strings. A generalized suffix tree allows finding substrings that are common to an ensemble of strings.

The nodes on the path from the root of the tree to a node *i *are the ancestors of *i*. The lowest common ancestor of nodes *i *and *j *is the furthest node from the root of the tree that is a common ancestor of both *i *and *j*. The string length of the path from the root of the tree to that node is the longest common extension of the suffixes *i *and *j*.

Briefly, a suffix array for a text *T *= *t*_1 _... *t*_*n *_is an array of integers that specifies the lexicographic order of the suffixes of *T; *each entry of this array is the start position of a suffix of *T*. This simple data structure is enhanced by pre-calculating other indexing structures in order to perform the top-down and bottom-up traversal, as well as calculating the longest common prefix.

### Data

All the 3' UTR entries containing the keyword histone as well as an HSL3 feature were extracted from UTRdb release 19 [32]. A total of 28 sequences was obtained. Each sequence contains an occurrence of the motif. The specific location of the motif is known and used in the calculation of the performance measures only. The remaining structure is unknown; see Section Performance measures for a detailed discussion of the evaluation of the results. The length of the sequences varies from 51 to 1,955 nucleotides, with an average length of 701 nucleotides. The dataset consists of the following entries: 3HSA054868, 3HSA041812, 3HSA027954, 3HSA034695, 3HSA079397, 3HSA082131, 3HSA047510, 3HSA083260, 3HSA083338, 3HSA083659, 3HSA048427, 3HSA049188, 3HSA084501, 3HSA086570, 3HSA086915, 3HSA087013, 3HSA089561, 3HSA058723, 3HSA058724, 3MMU017942, 3MMU040716, 3MMU043604, 3MMU045939, 3MMU046704, 3MMU004991, 3MMU004994, 3MMU004995 and 3DRE005245.

All the mammalian 5' UTR entries containing the keyword ferritin and a valid IRE motif were extracted from UTRdb release 19 [32]. A total of 14 sequences was obtained. Each sequence contains an occurrence of the motif. The specific location of the motif is known and used in the *a posteriori *analysis only. The remaining structure is unknown; see Section Performance measures for a detailed discussion of the evaluation of the results. The length of the sequences varies from 58 to 2,188 nucleotides, with an average length of 378 nucleotides. The dataset consists of the following entries: 5DLE000003, 5HSA021933, 5HSA033035, 5HSA060296, 5HSA072191, 5HSA073036, 5HSA079314, 5MMU018600, 5MMU025452, 5MMU027798, 5MMU032372, 5RNO004780, 5RNO005974 and 5RNO007816.

A tRNA dataset was assembled using a subset of the sequences from Masoumi and Turcotte [16]. Seven sequences having approximately the same length were used. These are generally challenging sequences for traditional approaches, such as MFOLD, see Table 4. The secondary structure description for the following entries were extracted from the compilation by Sprinzl *et al*. [33,34]: RD0260, RD0500, RD1140, RD2640, RE2140, RE6781 and RF6320.

Similarly, a 5S dataset was assembled using a subset of the sequences from Masoumi and Turcotte [16]. Seven sequences having approximately the same length were used. These are also generally challenging sequences for traditional approaches, such as MFOLD, see Table 5. The secondary structure description for the following entries were extracted from the Comparative RNA Web Site [35-37]: V00336, X02627, X04585, M24839, X67579, AJ251080 and M25591.

### Performance measures

We call **references**, the secondary structures that were obtained from curated databases such as the tRNA compilation by Sprinzl and the Comparative RNA Web Site. We define as **true positives **(TP) the base pairs that are occurring in both structures, reference and predicted, **false positives **(FP), the base pairs that are occurring in the predicted structure but not in the reference one, and **false negatives **(FN), the base pairs that are occurring in the reference structure but not in the predicted one. Offsets were not allowed.

The **positive predictive value **(PPV) is defined as the fraction of the predicted base pairs that are also present in the reference structure, TP/(TP + FP). The **sensitivity **is defined as the fraction of the base pairs from the reference structure that are correctly predicted, TP/(TP + FN). Finally, we also measured the **Matthews Correlation Coefficient**, as defined by Gorodkin, Stricklin and Stormo [38]:

TP(TP+FN)×TP(TP+FP)
 MathType@MTEF@5@5@+=feaafiart1ev1aaatCvAUfKttLearuWrP9MDH5MBPbIqV92AaeXatLxBI9gBaebbnrfifHhDYfgasaacH8akY=wiFfYdH8Gipec8Eeeu0xXdbba9frFj0=OqFfea0dXdd9vqai=hGuQ8kuc9pgc9s8qqaq=dirpe0xb9q8qiLsFr0=vr0=vr0dc8meaabaqaciaacaGaaeqabaqabeGadaaakeaadaGcaaqaamaalaaabaGaeeivaqLaeeiuaafabaGaeiikaGIaeeivaqLaeeiuaaLaey4kaSIaeeOrayKaeeOta4KaeiykaKcaaiabgEna0oaalaaabaGaeeivaqLaeeiuaafabaGaeiikaGIaeeivaqLaeeiuaaLaey4kaSIaeeOrayKaeeiuaaLaeiykaKcaaaWcbeaaaaa@41EE@

There can be more than one occurrence of a motif in a given sequence. During the discovery process, the selection of an occurrence is made by the scoring functions, for instance, *TLeft *selects the leftmost occurrence. In the evaluation of the results, the PPV of all the occurrences of a motif in a given sequence are computed. For each sequence, the occurrence with the highest PPV is selected. The selected occurrences are used to compute the average PPV; these are reported in the Experiments section. Similarly, other measures, sensitivity and MCC, are calculated for the same selected occurrences. In the case of UTRs, the secondary structure outside of the region containing the known motif is considered unknown. Any occurrence of a motif outside of the region of the known motif is scored zero. When computing the performance indices for MFOLD, the default parameters were used. If there were more than one prediction, the prediction with the best PPV was used.

## Parameters

For all the experiments, the minimum support is set to 70%, the minimum total number of base pairs is 5, G:U base pairs were allowed, no mismatches were allowed, and no time limit was set. Table 6 shows the parameters that vary between experiments.

## Authors' contributions

MA carried out ranking based experiments for his M.Sc. under the supervision of MT. TN coded the initial version of Seed for his M.Sc. under the supervision of MT. MT developed the overall design of this approach and worked on the implementation of the version of Seed currently available on the web. All the authors have read and approved the final manuscript.

## Figures and Tables

**Figure 10:**
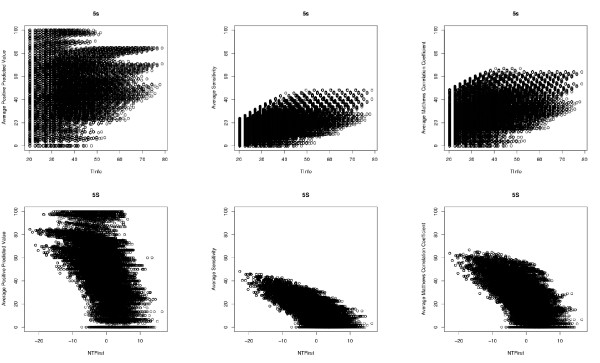
**Performance diagrams for the 5S rRNA experiment**. *TInfo *(top) and *NTFirst *(bottom) scores against the PPV, sensitivity and Matthews Correlation Coefficient for the 5S dataset.

**Table 1:** Runtime statistics. Details of the execution of Seed for all the 6 experiments showing the total number of motifs discovered (Motifs), the total number of structurally distinct motifs (Distinct), the number of matches made (Matches), the space and time taken for each run. The run time is composed of 1) construction of tree, 2) motif generation (search space) and 3) general to specific search of search space. Length shows the minimum and maximum length of the sequences in the corresponding dataset. Suffix of 01 and 02 indicate a different setup used on the same dataset. Finally, Sequences shows the number of sequences present in the dataset. These experiments were carried out on a Sun Fire V20z computer, 2 processor server, AMD Opteron 248 (2.2 GHz), 8 Gigabytes, Solaris 9, a single processor was used.

**Id**	**Sequences**	**Length**	**Motifs**	**Distinct**	**Matches**	**Space**	**Time**
HSL3 01	28	51–1,955	65	2	2,016	1.83 Mbytes	< 1 s
IRE 01	14	58–2,188	32	29	42,462	0.39 Mbytes	5 s
HSL3 02	28	51–1,955	357	232	1,945,328	1.37 Mbytes	5 m 21 s
IRE 02	14	58–2,188	110	102	167,076	0.46 Mbytes	25 s
tRNA	7	76–77	5,518	2,010	3,407,012	9.40 Mbytes	6 m 11 s
5S	7	117–120	364,505	24,645	152,741,463	0.52 Gbytes	7 h 40 m

**Table 2:** Correlation results. For each column, the numbers represent the correlation coefficient with the average Matthews Correlation Coefficient. Bold values correspond to the highest correlation with MCC.

**Score**	**HSL3 01**	**HSL3 02**	**IRE 01**	**IRE 02**	**tRNA**	**5S**	**Average**
**TInfo**	0.337	0.849	0.681	0.723	0.663	0.519	0.629
**TAvg**	-0.868	-0.964	-0.857	-0.626	-0.792	-0.680	-0.798
**TBest**	-0.385	-0.960	-0.869	-0.608	-0.829	-0.755	-0.734
**TFirst**	-0.465	-0.970	**-0.891**	-0.673	-0.827	-0.743	-0.762
**TLeft**	-0.529	-0.970	-0.869	-0.638	-0.831	-0.744	-0.764
**TSum**	-0.483	-0.950	-0.840	-0.656	-0.785	**-0.764**	-0.746
**TWorst**	-0.561	-0.973	-0.880	-0.661	-0.825	-0.735	-0.773
**NTAvg**	-0.450	-0.973	-0.836	-0.644	-0.797	-0.679	-0.730
**NTBest**	-0.879	-0.966	-0.857	-0.610	-0.830	-0.754	-0.816
**NTFirst**	-0.776	-0.974	-0.887	-0.682	-0.830	-0.743	-0.815
**NTLeft**	**-0.959**	-0.976	-0.860	-0.647	**-0.834**	-0.744	**-0.837**
**NTSum**	-0.865	-0.948	-0.841	-0.663	-0.784	-0.757	-0.810
**NTWorst**	-0.740	**-0.978**	-0.880	**-0.685**	-0.828	-0.734	-0.808

**Table 3:** Rank statistics. Ranking statistics ρ^
 MathType@MTEF@5@5@+=feaafiart1ev1aaatCvAUfKttLearuWrP9MDH5MBPbIqV92AaeXatLxBI9gBaebbnrfifHhDYfgasaacH8akY=wiFfYdH8Gipec8Eeeu0xXdbba9frFj0=OqFfea0dXdd9vqai=hGuQ8kuc9pgc9s8qqaq=dirpe0xb9q8qiLsFr0=vr0=vr0dc8meaabaqaciaacaGaaeqabaqabeGadaaakeaaiiGacuWFbpGCgaqcaaaa@2E83@ for the objective functions for all six experiments. MCC has been used as the response variable. Bold values indicate the highest value of ranking statistics.

**Score**	**HSL3 01**	**HSL3 02**	**IRE 01**	**IRE 02**	**tRNA**	**5S**	**Average**
**TInfo**	0.99392	0.75403	0.74010	0.50895	0.68576	0.46454	0.69122
**TAvg**	**1.0**	0.91768	0.93878	0.70182	0.84418	0.66779	0.84504
**TBest**	0.95940	0.92308	**0.94978**	0.71150	0.87339	0.74767	0.86080
**TFirst**	0.97946	0.89990	0.93072	0.77785	0.87396	0.73486	0.86613
**TLeft**	0.95940	0.91821	0.88710	0.72931	0.87673	0.73630	0.85118
**TSum**	0.96206	0.86379	0.89809	0.74273	0.85686	0.76647	0.84833
**TWorst**	0.99790	0.90865	0.89919	0.72699	0.87211	0.72388	0.85479
**NTInfo**	0.97487	0.56468	0.58651	0.32658	0.60901	0.45417	0.58597
**NTAvg**	0.97692	0.92258	0.92595	0.71508	0.84540	0.66690	0.84214
**NTBest**	**1.0**	**0.92592**	0.93878	0.71489	0.87308	**0.74639**	0.86651
**NTFirst**	0.99939	0.90302	0.92375	**0.78037**	0.87416	0.73371	**0.86907**
**NTLeft**	**1.0**	0.92229	0.88453	0.75067	**0.87686**	0.73515	0.86158
**NTSum**	0.99843	0.90842	0.94318	0.74071	0.87643	0.73789	0.86751
**NTWorst**	0.99825	0.91239	0.85887	0.75025	0.87272	0.72281	0.85255

**Table 4:** The tRNA dataset. Base pairs from the top-ranked (*NTFirst*) motif were used as structural constraints for MFOLD. The table presents the PPV and sensitivity for the application of MFOLD alone as well as the combined approach onto the tRNA dataset. The number in between parentheses represents the number of sub-optimal structures reported with similar performance measures.

**Id**	**Method**	**% PPV**	**% Sensitivity**
RD0260	MFOLD (2)	28.6–29.2	28.6–33.3
	MFOLD (1)	66.7	57.1
	MFOLD (1)	57.1	57.1
	**Seed NTFirst + MFOLD**	**100.0**	**100.0**
RD1140	MFOLD (1)	100.0	100.0
	**Seed NTFirst + MFOLD**	**100.0**	**100.0**
RD2640	MFOLD (1)	63.6	66.7
	MFOLD (2)	18.2	19.0
	**Seed NTFirst + MFOLD**	**100.0**	**100.0**
RE2140	MFOLD (1)	87.0	95.2
	MFOLD (1)	69.6	76.2
	**Seed NTFirst + MFOLD**	**91.3**	**100.0**
RE6781	MFOLD (4)	28.0–31.8	33.3
	**Seed NTFirst + MFOLD**	**100.0**	**100.0**

**Table 5:** The 5S rRNA dataset. Base pairs from the top-ranked (*NTFirst*) motif were used as structural constraints for MFOLD. The table presents the PPV and sensitivity for the application of MFOLD alone as well as the combined approach onto the 5S rRNA dataset. Two setups were used for the combined approach. First, the top-ranked motif, according to *NTFirst*, was used. Second, a high PPV motif was selected to illustrate the expected benefit for the combined approach. Numbers in between parentheses indicate the number of sub-optimal structures reported with similar performance measures.

**Id**	**Method**	**% PPV**	**% Sensitivity**
V00336	MFOLD (2)	25.0	25.6–26.3
	Seed NTFirst + MFOLD	81.1	75.0
	Seed Best PPV + MFOLD	62.5	67.6
	**Seed Best PPV + MFOLD**	**92.5**	**100.0**
X02627	MFOLD (1)	30.8	32.4
	MFOLD (1)	84.6	89.2
	Seed NTFirst + MFOLD	75.7	71.8
	**Seed Best PPV + MFOLD**	**89.7**	**89.7**
X04585	MFOLD (1)	23.7	25.0
	MFOLD (11)	18.4	20.0–23.3
	Seed NTFirst + MFOLD	60.0	55.3
	**Seed Best PPV + MFOLD**	**73.7**	**77.8**
AJ251080	MFOLD (1)	65.8	73.5
	MFOLD (1)	60.5	65.7
	MFOLD (2)	42.1	42.1–43.2
	MFOLD (1)	55.3	67.7
	Seed NTFirst + MFOLD	66.7	63.2
	**Seed Best PPV+ MFOLD**	**71.1**	**75.0**
	Seed Best PPV + MFOLD	65.8	67.6
M25591	MFOLD (1)	71.1	77.1
	MFOLD (2)	42.1	43.2–44.4
	MFOLD (1)	60.5	71.9
	Seed NTFirst + MFOLD	70.6	63.2
	**Seed Best PPV + MFOLD**	**76.3**	**78.4**

**Table 6:** Parameters settings. Parameters settings for each experiment: stem_min_len is the minimum length of complementary regions that are reported in the first step of the algorithm, min_num_stem specifies the minimum number of complementary segments each motif should have to be part of the output, max_num_stem is a stopping criteria, the algorithm does not extend motifs beyond this threshold, stem_max_separation is the maximum distance between the start and the end positions of a helix, finally, range is the number of allowed insertions allowed by the pattern matcher.

**Experiment**	**stem_min_len**	**mim_num_stem**	**max_num_stem**	**stem_max_separation**	**range**
HSL3 01	6	1	1	30	0
HSL3 02	3	1	-	30	0
IRE 01	3	1	2	30	0
IRE 02	3	1	-	30	0
tRNA	3	3	-	-	1
5S	3	3	-	-	1
